# Physicochemical Characterization of Injectable Genipin-Crosslinked Gelatin–Kelulut Honey Hydrogels for Future Cutaneous Tissue Loss

**DOI:** 10.3390/polym17091129

**Published:** 2025-04-22

**Authors:** Raniya Razif, Nur Izzah Md Fadilah, Manira Maarof, Daniel Looi Qi Hao, Adzim Poh Yuen Wen, Mh Busra Fauzi

**Affiliations:** 1 Department of Tissue Engineering and Regenerative Medicine (DTERM), Faculty of Medicine, Universiti Kebangsaan Malaysia, Cheras, Kuala Lumpur 56000, Malaysia; raniyarazif@gmail.com (R.R.); izzahfadilah@ukm.edu.my (N.I.M.F.); manira@ukm.edu.my (M.M.); 2 Advance Bioactive Materials-Cells UKM Research Group, Universiti Kebangsaan Malaysia, Bangi 43600, Selangor, Malaysia; 3 My Cytohealth Sdn Bhd, Hive 5, Taman Teknologi, MRANTI, Bukit Jalil, Kuala Lumpur 57000, Malaysia; dr.daniellooi@cytoholdings.com; 4 Department of Surgery, Hospital Canselor Tuanku Muhriz, Universiti Kebangsaan Malaysia, Cheras, Kuala Lumpur 56000, Malaysia; dradzimpoh.hctm@ukm.edu.my

**Keywords:** injectable hydrogel, bioinks, 3D bioprinting, gelatin, Kelulut honey, skin, wound healing

## Abstract

Chronic wounds, particularly those associated with conditions like diabetes, present significant challenges in healthcare due to prolonged healing and high susceptibility to infections. This study investigates the development of injectable hydrogels composed of genipin-crosslinked gelatin and Kelulut honey (KH) as novel biomaterials for wound healing applications. Hydrogels were prepared with varying concentrations (*w*/*v*) of gelatin (9% and 10%) and KH (0.1% and 0.5%), with genipin (0.1%) acting as a crosslinker. The physicochemical properties were extensively evaluated, including the swelling ratio, water vapor transmission rate (WVTR), contact angle, porosity, enzymatic degradation, and surface roughness. The results showed that KH incorporation significantly enhanced the swelling properties of the hydrogels, with the 9GE_0.1KH formulation demonstrating a swelling ratio of 742.07 ± 89.61% compared to 500% for the control 9GE formulation. The WVTR values for KH-incorporated hydrogels ranged from 1670.60 ± 236.87 g/m^2^h to 2438.92 ± 190.90 g/m^2^h, which were within the ideal range (1500–2500 g/m^2^h) for wound healing. Contact angle measurements indicated improved hydrophilicity, with 9GE_0.1KH showing a contact angle of 42.14° ± 7.52° compared to 60° ± 11.66° for the 10GE formulation. Biodegradation rates were slightly higher for KH-modified hydrogels (0.079 ± 0.006 mg/h for 9GE_0.1KH), but all remained within acceptable limits. These findings suggest that genipin-crosslinked gelatin-KH hydrogels offer a promising scaffold for enhanced wound healing and potential applications in tissue engineering and three-dimensional (3D) bioprinting technologies.

## 1. Introduction

The skin serves as the primary protective barrier for the body, safeguarding it against pathogens and physical injuries while also help regulating temperature, water content, and electrolyte balance [1,2]. The skin also maintains physiological hemostasis through immune–neuroendocrine functions [2,3]. Wounds are injuries to the skin tissues that result from trauma, burns, or disease, and can be classified as acute or chronic based on the healing time. Acute wounds typically heal quickly, while chronic wounds, which are normally associated with conditions like diabetes, obesity, and vascular diseases, take longer and may become life-threatening if not managed properly [4].

Cutaneous tissue loss refers to the loss of skin and sometimes underlying tissues due to various causes that are similar to wounds; primarily, chronic wounds can lead to significant physiological imbalances and potential disability [5,6]. Cutaneous tissue loss in patients with diabetes is closely linked to diabetes-related complications. Poor glycemic control contributes to microvascular issues like neuropathy, retinopathy, and nephropathy, which impair skin integrity and healing. Patients with diabetes are also more susceptible to infections, such as cutaneous candidiasis, which worsen tissue loss due to weakened immune responses, including reduced macrophage activity and altered cytokine production, leading to delayed healing and further damage. Cutaneous tissue loss is a major healthcare challenge, affecting 8.5 million people in the U.S. and costing USD 28–90 billion annually [7,8]. It severely impacts patients’ physical, mental, and social well-being, with chronic wounds linked to psychological symptoms that reduce quality of life, particularly in daily activities and social interactions [9].

Traditional wound care methods, such as debridement, skin flaps, grafts, and non-surgical treatments like antiseptics and dressings, are commonly used for both acute and chronic wounds [10,11]. However, their efficacy is often limited in chronic wounds, requiring supplementary treatments like special programs to improve healing [12,13]. Tissue engineering, which involves bioactive materials, stem cells, and skin substitutes, offers an alternative by enhancing tissue repair and minimizing scarring [14]. Integrating 3D bioprinting technology allows for more customized solutions using injectable hydrogels and bioinks to support tissue regeneration and angiogenesis [15]. Three-dimensional bioprinting plays a crucial role in creating customizable, biocompatible scaffolds that replicate the natural extracellular matrix (ECM), promoting tissue integration and regeneration [16,17]. Using techniques like extrusion and laser-assisted bioprinting, it is possible to create scaffolds with specific micro- and macro-scale features that support multifunctional properties, such as hemostasis and anti-inflammatory and antibacterial effects [18]. Bioinks made from biomaterials, live cells, and bioactive compounds are applied layer by layer to form 3D scaffolds, improving their functionality and better mimicking the dynamic nature of native tissues [19,20].

Gelatin, derived from fish, bovine, and porcine collagen, is a versatile biopolymer valued for its biocompatibility, non-immunogenicity, and modifiable properties, making it suitable for drug delivery and tissue engineering [21]. Gelatin-based hydrogels, such as GelMA, mimic soft tissue mechanics and promote wound healing [22,23]. These hydrogels are cost-effective, derived from by-products like skin and bones, and possess non-toxicity, biodegradability, and biocompatibility. Genipin, a natural crosslinker, enhances gelatin’s mechanical, thermal, and biological properties, improving the tensile strength, microhardness, and enzymatic resistance of 3D bioprinted scaffolds, while reducing cytotoxicity and improving swelling properties [24,25,26,27,28,29]. Kelulut honey, produced by stingless bees, offers potent antimicrobial, anti-inflammatory, and antioxidant properties, accelerating wound healing and promoting dermal repair, epithelialization, and granulation tissue formation compared to normal honey from honeybees due to its distinct composition and physicochemical properties [30,31,32,33]. Kelulut honey has a higher moisture content, varying from 25–35%, compared to normal honey, which typically has a 17–20% moisture content, helping maintain a moist wound environment essential for wound healing [30]. This higher moisture content not only promotes epithelialization and accelerates tissue regeneration but also enhances the bioavailability of its antimicrobial and antioxidant compounds, optimizing their efficacy in reducing infections and supporting dermal repair. Its antioxidant and antimicrobial effects, coupled with moisture retention, aid in infection reduction and healing [32,33,34,35,36]. Studies confirm its antibacterial efficacy, with a minimum inhibitory concentration of 5–12.5%, and it has demonstrated safety in subacute oral toxicity studies [37,38].

While previous studies have explored honey–gelatin composites, they have primarily focused on conventional honey types combined with other bioactive compounds. In contrast, this study specifically investigates the physicochemical and bioactive properties of Kelulut honey within a gelatin–genipin-based hydrogel system. Example of previous studies would be the usage of Manuka honey by Tomic et al. in 2023 and thyme honey by Lahooti et al. in 2016 for wound healing application [39,40]. Both studies have different compositions of honey–gelatin hydrogels with an investigation of different aspects of the gelatin–honey composites. In contrast, this study specifically investigates the physicochemical and bioactive properties of Kelulut honey within a gelatin–genipin-based hydrogel system. Given Kelulut honey’s unique promotion of cell viability, polymerization time, physicochemical properties, mechanical properties, chemical and structural composition, and 3D bioprinting capability, its incorporation into a gelatin–genipin matrix may yield distinct advantages in wound healing applications. The combination of genipin as a crosslinker, known for its biocompatibility and ability to enhance mechanical properties, with the unique properties of Kelulut honey sets this study apart.

**Figure 1:**
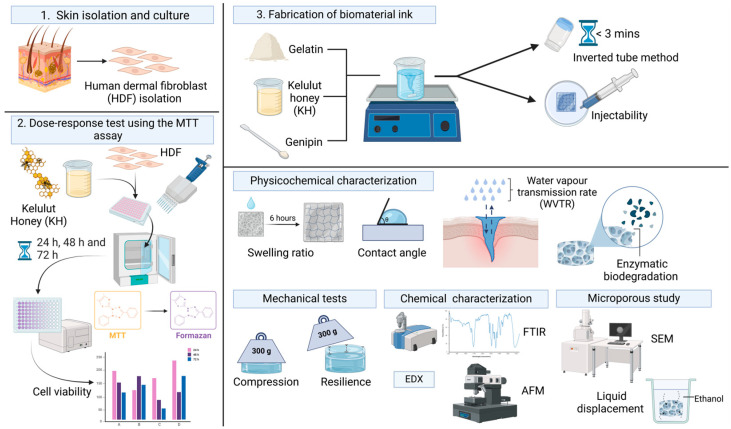
Flow of the study of the injectable gelatin–Kelulut honey hydrogel. Images were created using https://BioRender.com (accessed on 15 January 2025).

The combination of gelatin, genipin, and Kelulut honey into a single bioink provides significant advantages for bioprinting and tissue engineering. Genipin improves the bioink’s mechanical properties and biodegradability, while Kelulut honey adds antimicrobial protection, supporting cell growth and differentiation. This synergy results in a material with tailored degradation rates that is ideal for advanced tissue engineering and regenerative medicine applications [24,28,41]. In this study, an injectable hydrogel composed of genipin-crosslinked gelatin and Kelulut honey was developed to promote skin healing, with the aim of evaluating its physicochemical and cytotoxic properties for wound care applications, as shown in Figure 1. The incorporation of Kelulut honey was expected to enhance bioactivity, while genipin optimization aimed to improve mechanical stability and biocompatibility, making the hydrogel a promising candidate for skin tissue regeneration and 3D bioprinting.

## 2. Study Design

The study design was approved by the Universiti Kebangsaan Malaysia Research Ethics Committee (Code noJEP-2024-904) with research grant under Geran Fundamental Fakulti Perubatan from Faculty of Medicine, Universiti Kebangsaan Malaysia (Grant code: FF-2024-447).

### 2.1. Skin Cell Isolation and Culture

**Figure 2:**
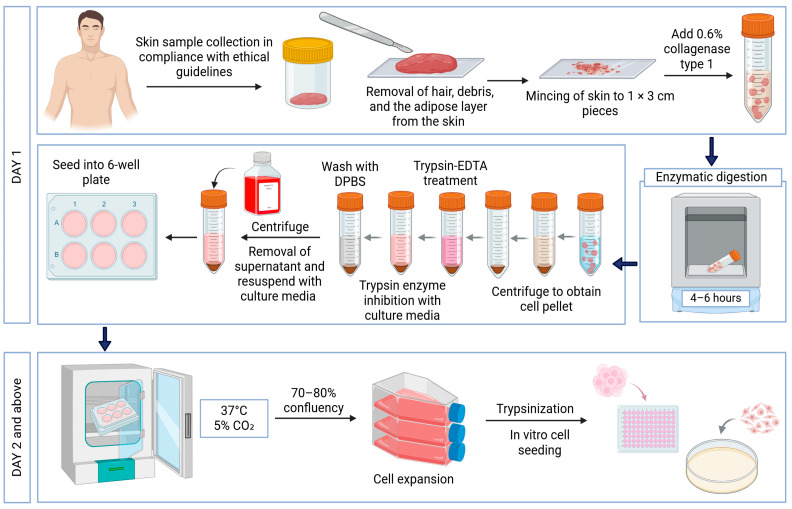
Experimental design for human skin cell isolation. Images were created using https://BioRender.com (accessed on 21 February 2025).

Primary human dermal fibroblasts (HDFs) were isolated from excess skin tissue obtained from consenting patients. Skin samples were processed using 0.6% collagenase type I (Worthington-Biochemical Corporation, 730 Vassar Ave Lakewood, NJ, USA) and trypsin-EDTA (Gibco, Carlsbad, CA, USA), with Dulbecco’s phosphate buffered saline (DPBS; Gibco, Carlsbad, CA, USA) washes, followed by centrifugation and resuspension in Dulbecco’s modified Eagle’s medium/nutrient mixture F12 (DMEM/F12) supplemented with 10% fetal bovine serum (FBS; Gibco/BRL, Carlsbad, CA, USA). Cells were seeded in 6-well plates, cultured until 70–80% confluence, and then subcultured. Expanded HDFs were maintained in flasks containing DMEM/F12 with 10% FBS. The experimental workflow is illustrated in Figure 2.

### 2.2. Dose–Response Test

The impact of Kelulut honey (Kuching, Sarawak, Malaysia) on cell viability was evaluated using the MTT (3-[4,5-dimethylthiazol-2-yl]-2,5 diphenyl tetrazolium bromide) assay. Cells were seeded in 48-well plates (10,000 cells/well) and incubated for 24 h. Subsequently, cells were exposed to varying Kelulut honey concentrations (0.1%, 0.5%, 1.0%, 4.0%, and 10.0%) for 24, 48, or 72 h. Following each treatment, MTT solution (10%) was added to each well, and the plates were incubated for 4 h at 37 °C. After 4 h, the MTT solution was removed, and DMSO (dimethyl sulfoxide) was added. The plates were incubated for 10 min. The dissolved formazan solution was transferred to a 96-well plate, and the absorbance was measured at 540 nm using a microplate reader. Absorbance readings are proportional to the number of viable cells.

### 2.3. Hydrogel Preparation

Gelatin solutions (9% and 10% *w*/*v*) were prepared by dissolving gelatin powder (Nitta-Gelatin Ltd., Yao City, Osaka, Japan) in distilled water (dH_2_O) at 40 °C with stirring. KH honey solutions (0.1%, 0.5%, 1.0%, 4.0%, and 10.0% *v*/*v*) were separately prepared in dH_2_O and then incorporated into the gelatin solutions, creating six formulations: 9GE_0.1KH, 9GE_0.5KH, 9GE_1.0KH, 10GE_0.1KH, 10GE_0.5KH, and 10GE_1.0KH. A 0.1% (*w*/*v*) genipin (FUJIFILM Wako Pure Chemical Corporation, Osaka, Japan) crosslinking solution was prepared in 70% ethanol (EtOH; MERCK, Darmstadt, Germany) and subsequently added to each gelatin–honey formulation.

### 2.4. Polymerization Time and Injectability

The polymerization process of the hydrogels was monitored at a temperature of 23 ± 2 °C, and the time required for complete gelation was recorded using the inverted tube test method.

### 2.5. Evaluation of the Gross Appearance

To assess the impact of Kelulut honey (KH) on the final hydrogel extrudability as a biomaterial ink, the appearance of crosslinked formulations, both with and without KH, was evaluated. Injectable hydrogels were evaluated by extruding the formulations through a syringe and nozzle. The gross morphology of the control hydrogels (9GE and 10GE) was compared to that of the KH-incorporated hydrogels (9GE_0.1KH and 10GE_1.0KH). Images were captured from top and cross-sectional views using a digital camera (Nikon, Tokyo, Japan).

### 2.6. Swelling Ratio

To assess the capacity of the hydrogels to absorb fluids, their swelling behavior was investigated using a method adapted from a previous study [29]. Freeze-dried hydrogels were first weighed (*Wi*) and then immersed in Dulbecco’s phosphate buffered saline (DPBS, pH 7.4) at room temperature for 6 h. After this incubation period, the excess DPBS was carefully removed using filter paper, and the hydrogels were weighed again to obtain their final weight (*Wf*). These data were then used to calculate the swelling ratio, which reflects the hydrogel’s ability to absorb and retain fluids, a crucial property for wound healing applications. The swelling ratio can be calculated using the following formula:

$$Swelling\;ratio(\%)=\frac{\left(Wf-Wi\right)}{Wf}\times 100$$

### 2.7. Porosity

The porosity analysis was conducted on freeze-dried hydrogels prepared using two different methods, as outlined below.

#### 2.7.1. SEM

To analyze the internal structure of the hydrogels, they were first freeze-dried and then coated with a thin layer of gold to enhance image quality. Following a previously established method [42], the hydrogels were then imaged using a high-resolution field-emission scanning electron microscope (FESEM, Supra 55VP model, Jena, Germany). The pore diameters were then analyzed and calculated using Image J software (V1.5, Bethesda, MD, USA).

#### 2.7.2. Liquid Displacement

A porosity analysis was performed through liquid displacement using ethanol as the displacement fluid. Due to its non-polar nature, ethanol does not interact with the polymeric network of the hydrogel, thus preventing swelling or deformation of the scaffold. Instead, ethanol effectively permeates the hydrogel matrix, occupying the interstitial spaces and providing an accurate measure of the total pore volume. Freeze-dried hydrogels were immersed in ethanol, and the percentage porosity was calculated based on the weight difference before and after immersion, according to the following equation:

$$Porosity(\%)=\frac{Wf-Wi}{pV}\times 100$$

In the equation, *Wf* represents the final weight of the scaffold after ethanol immersion, *Wi* denotes the initial weight of the scaffold, *ρ* is the density of ethanol (0.789 g/m^3^), and *V* signifies the volume of the scaffold.

### 2.8. Contact Angle

The hydrophilicity of the polymerized hydrogels was evaluated by measuring the contact angle formed by a 10 μL droplet of dH_2_O on the hydrogel surface. Images of the water droplets were captured with a digital camera and analyzed using ImageJ software (version 1.54k, NIH, Bethesda, MD, USA) to determine the contact angle.

### 2.9. Water Vapor Transmission Rate

The water vapor transmission rate (WVTR) of the hydrogels was evaluated according to the American Society for Testing and Materials (ASTM) standard [42,43] to evaluate their capability for moisture regulation and gas exchange, crucial factors in promoting wound healing. In this experiment, each hydrogel was placed on the mouth of a cylindrical jar containing 10 mL of dH_2_O. The assembly was then incubated at 37 °C in a controlled atmosphere with 5% CO_2_. The following equation was used to calculate the WVTR, where *Wi* represents the initial weight of the assembly, *Wf* denotes the final weight after the incubation period, and *A* is the surface area of the cylindrical cup:

$$WVTR=\frac{\left(Wf-Wi\right)}{\left(A\times Time\right)}$$

### 2.10. Enzymatic Degradation

To assess enzymatic degradation, hydrogels were weighed (*W1*) and subsequently immersed in a solution of 0.0006% (*w*/*v*) collagenase type I (Worthington, Lakewood, NJ, USA) within a 24-well plate. Following a 6 h incubation period at 37 °C, the collagenase solution was removed, and the hydrogels were rinsed with dH_2_O to eliminate the residual enzyme. The hydrogels were then subjected to a second weighing (*W2*) to determine the mass remaining after enzymatic degradation. The percentage weight loss, indicative of the extent of biodegradation, was calculated using the following equation:

$$Weight\;Loss(\%)=\frac{(W2-W1)W1}{W1}\times 100$$

### 2.11. Mechanical Testing

#### 2.11.1. Compression

The mechanical properties of the hydrogels were evaluated using a modified compression test adapted [42,44]. Cylindrical hydrogel samples, approximately 2 cm in diameter and 3 mm in height, were subjected to compression at room temperature. The compressive modulus (*E*), a measure of the hydrogel’s stiffness, was determined using the following formula [44,45]:

$$E=\frac{\sigma }{\varepsilon }$$

*σ* = compressive force per unit area (stress)
*ε* = changes in volume per unit volume (strain)

#### 2.11.2. Resilience

The resilience of the hydrogels, or their ability to recover their original shape after compression, was evaluated using a modified method adapted from [42]. A 300 g metal load was applied to each hydrogel for 5 min. Images of the hydrogels were captured before and after compression using a digital camera equipped with a scale to ensure accurate measurements. Changes in hydrogel thickness were then analyzed using ImageJ software (version 1.54k, NIH, Bethesda, MD, USA). The percentage resilience (R) was calculated using the following equation:

$$Resilience(\%)=\frac{Ai}{Af}\times 100$$

where *Ai* is the area of thickness area before compression and *Af* represents the thickness after the compression, indicating the hydrogel’s ability to revert to its original size.

### 2.12. Surface Roughness

The surface roughness of the freeze-dried hydrogels was analyzed using atomic force microscopy (AFM) (Park Systems NX-10 instrument, Suwon, Republic of Korea). Images were obtained in non-contact mode at a scan rate of 0.2 Hz, with a scan size of 5 × 2 nm and a resolution of 256 × 256 pixels. The resulting AFM images were processed using the XE Image Processing Program to quantify the surface roughness of the 5 × 5 mm hydrogel samples.

### 2.13. Sample Characterization

#### 2.13.1. FTIR Analysis of the Hydrogel’s Functional Groups

To investigate the chemical structure of the hydrogels and any alterations induced by crosslinking or the incorporation of water-soluble Kelulut honey, Fourier transform infrared (FTIR) spectroscopy was utilized. A Perkin Elmer spectrometer (Waltham, MA, USA) was used to acquire spectra over a wavenumber range of 4000 cm^−1^ to 500 cm^−1^, enabling the identification of characteristic functional groups through their absorbance peaks.

#### 2.13.2. EDX

Furthermore, an energy-dispersive X-ray (EDX) analysis was carried out to assess the elemental composition of the hydrogels. A Phenom Pro X SEM EDX microscope (Phenom, Eindhoven, The Netherlands) was used to perform this analysis. The controls for this step were commercial gelatin, genipin powder, and Kelulut honey.

### 2.14. Statistical Analysis

The statistical analysis of the data was performed using GraphPad Prism (V10.0, GraphPad Software Inc., San Diego, CA, USA). One-way and two-way ANOVA were employed to assess significant differences between multiple groups. All quantitative data are presented as means ± standard deviations, and statistical significance was determined using a *p*-value < 0.05. All measurements and experiments were obtained from three independent replicates (N = 3).

## 3. Results

### 3.1. Dose–Response Test with Kelulut Honey

**Figure 3:**
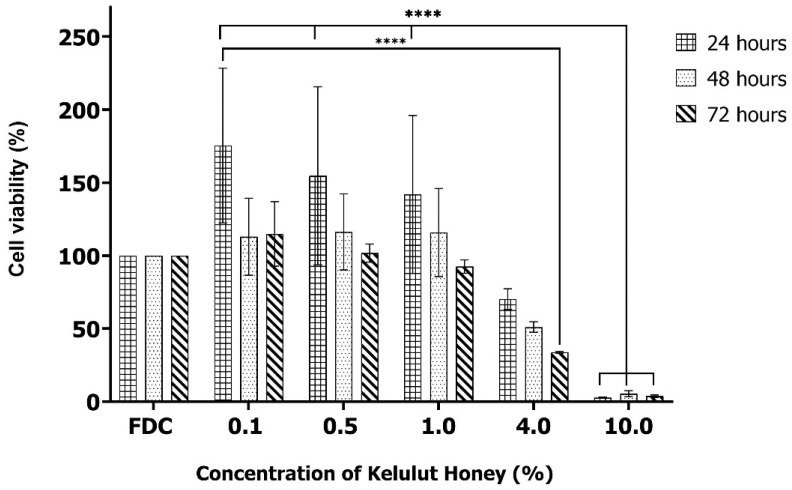
Cytotoxicity of different concentrations of Kelulut honey (0.1%, 0.5%, 1.0%, 4.0%, and 10.0%) toward human dermal fibroblasts measured using the MTT assay. FDC represents the control media used in the MTT assay. **** Indicates *p* < 0.0001.

Figure 3 illustrates the viability of fibroblasts following exposure to varying concentrations of Kelulut honey (0.1%, 0.5%, 1.0%, 4.0%, and 10.0%) for 24, 48, or 72 h. At lower concentrations of Kelulut honey (0.1%, 0.5%, and 1.0%), cell viability remained relatively high, often surpassing 100% at 24 and 48 h. This suggests that lower concentrations may promote cell proliferation or do not have any cytotoxic effect. Among these, the 0.1% concentration resulted in the highest viability, especially at the 24 h time point. In contrast, at 4.0%, cell viability dropped significantly, indicating a cytotoxic threshold. The most severe cytotoxic effect was observed at 10.0%, where cell viability approached 0% at all time points, confirming that high concentrations of Kelulut honey were detrimental to fibroblasts. A significant reduction in cell viability was observed at the highest concentration (10.0%) compared to the 0.1%, 0.5%, and 1.0% concentrations at 24 h, as indicated by the significant difference marker (****, *p* < 0.0001) and the decrease in the percentage of viable cells. There was also a significant reduction in the viability of the fibroblasts for a 4.0% concentration of Kelulut honey at the 72 h time point compared to the 0.1% concentration at the 24 h time point. This strong dose-dependent cytotoxic effect suggests that while Kelulut honey may have beneficial or non-cytotoxic effects at low concentrations, exceeding a certain threshold results in harmful consequences for fibroblast survival. Additionally, the trend across all concentrations revealed a general decrease in cell viability with increasing incubation time, highlighting the cumulative cytotoxic effects of Kelulut honey after longer exposures.

### 3.2. Gross Appearance and Polymerization Time

**Figure 4:**
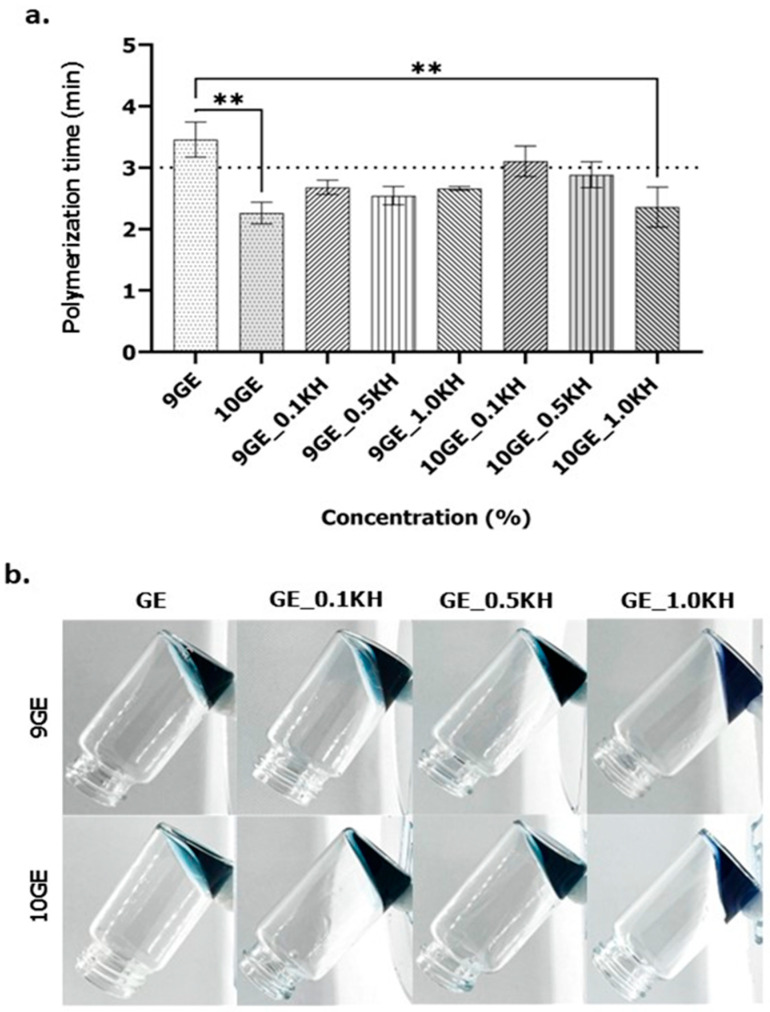
(**a**). The polymerization times for 9GE and 10GE hydrogels with and without Kelulut honey (** indicates *p* < 0.01). (**b**). The inverted jar method to analyze the polymerization time of 9GE and 10GE hydrogels with and without Kelulut honey.

The graph in Figure 4a illustrates the effects of various biomaterial ink formulations on the polymerization time. While a higher polymer concentration generally corresponds to faster polymerization, the interaction with Kelulut honey (KH) reveals a more complex relationship. Specifically, formulations with 0.1KH exhibit significantly slower polymerization, whereas those with 1.0KH demonstrate the most rapid polymerization, regardless of the base polymer (9GE or 10GE). Interestingly, all formulations containing KH achieve polymerization within the critical 3 min threshold, except for 10GE_0.1KH, which has the lowest KH concentration, highlighting the potential of KH addition at higher concentrations to accelerate the process. This suggests that the KH concentration plays a critical role in optimizing the polymerization time, potentially exceeding the influence of the polymer concentration alone.

Figure 4b presents the results of the inverted tube assay, visualizing the polymerization of different concentrations of gelatin incorporating Kelulut honey (KH) and 0.1% genipin crosslinking. The images illustrate the samples after a set period in the inverted position, providing a qualitative assessment of gel formation.

**Figure 5:**
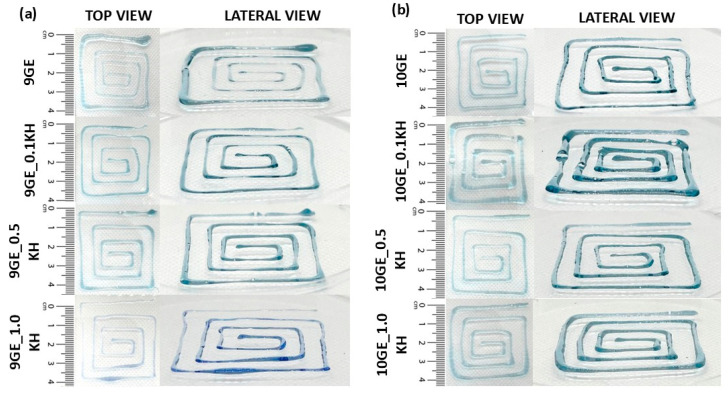
Gross appearance (top and lateral view) of (**a**) 9 GE and (**b**) 10 GE hydrogels with and without Kelulut honey.

Figure 5 presents the injectability of all ink formulations from both the top and lateral views. The inks exhibit a light blue or teal hue and display a gel-like or viscous consistency, as evidenced by their ability to maintain a spiral shape. Additionally, the materials appear translucent or semi-transparent. Notably, some formulations have a greater width than others.

### 3.3. Physicochemical Analysis

**Figure 6:**
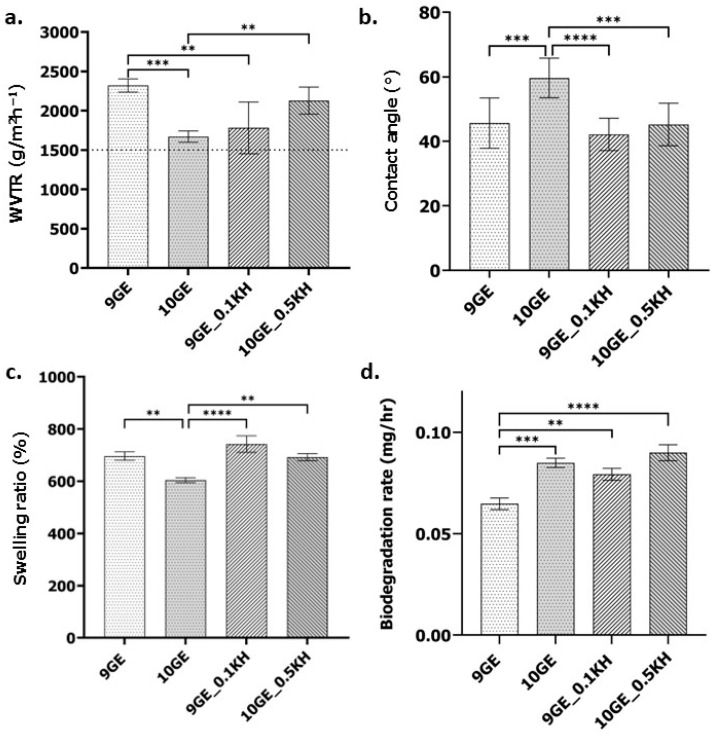
Physicochemical analysis of 9GE, 10GE, 9GE_0.1KH and 10GE_10KH. (**a**) Water vapor transmission rate (WVTR) (g/m^2^h^−1^), (**b**) contact angle (°), (**c**) percentage of swelling ratio, and (**d**) biodegradation rate. ** indicates *p* < 0.01, *** indicates *p* < 0.001, and **** indicates *p* < 0.0001.

The physicochemical properties of gelatin–KH hydrogels were evaluated by measuring the water vapor transmission rate (WVTR), contact angle, swelling ratio, biodegradation rate, and pore size. Figure 6 displays the results of a detailed examination of the quantitative data for KH-incorporated hydrogels with their non-KH-incorporated counterparts.

The water vapor transmission rate (WVTR) results in Figure 6a demonstrate dynamic relationships between the gelatin concentration, KH incorporation, and water vapor transmission. As shown in the bar graph, comparing different biomaterial formulations reveals that the 9GE formulation exhibits the highest WVTR, which is 2438.92 ± 190.90 g/m^2^h^−1^, significantly surpassing 10GE, as indicated by the *** (*p* < 0.001) statistical significance marker. The 10GE formulation shows a lower WVTR (1670.60 ± 236.87 g/m^2^h^−1^), suggesting reduced permeability. The addition of Kelulut honey (KH) at both 0.1% (9GE_0.1KH) and 0.5% (10GE_0.5KH) results in WVTR values that appear comparable to or slightly higher than 10GE. Specifically, the 9GE_0.1KH formulation shows a moderately significant difference compared to 9GE formulations, with the two asterisks ** (*p* < 0.01) indicating a significant reduction in the WVTR between 9GE_0.1KH and 9GE with the addition of KH. However, the significant difference between 10GE_0.1KH and 10GE_0.5KH shows a different observation, as the addition of KH shows a moderately significant difference, as can be seen with the ** *p* < 0.01; but, the addition of a higher concentration of KH significantly increases the WVTR of 10GE. This shows that the addition of KH and a higher concentration of KH affect the WVTR, as an increase in the KH concentration in the 10GE formulation increases the WVTR. Notably, all KH-incorporated hydrogels fall within the acceptable WVTR range of more than 1500 g/m^2^h^−1^, highlighting their potential for applications requiring controlled moisture transfer.

Figure 6b displays the contact angles for different gelatin and gelatin–KH hydrogel formulations. All formulations exhibited a contact angle below 90°, indicating a degree of hydrophilicity. The highest contact angle (60° ± 11.66°) was observed for the 10GE hydrogel. Notably, incorporating KH consistently lowered the contact angle. For instance, 9GE_0.1KH showed a contact angle of 42.14° ± 7.52°, while 10GE_0.5KH had a contact angle of 45.22° ± 9.41°. This suggests that KH enhances the hydrophilicity of the hydrogel surface. Furthermore, a comparison between 9GE and 10GE revealed a significant difference, with the 10GE formulation exhibiting a more hydrophobic nature, as the contact angle increased significantly, as can be seen with the *** (*p* < 0.001) statistical significance marker. The addition of Kelulut honey (KH), especially at higher concentrations, further decreased the contact angle, enhancing the hydrogel’s hydrophilic properties. The contact angle of 10GE was significantly higher than 9GE_0.1KH and can be seen by the high significance difference **** (*p* < 0.0001) statistical significance marker, demonstrating that the addition of 0.1% KH markedly enhanced hydrophilicity by reducing the contact angle. The contact angle of 10GE decreased significantly with the addition of 0.5% KH *** (*p* < 0.001), indicating a dose-dependent enhancement in the hydrophilicity of the hydrogel. These observations collectively indicate that while a higher gelatin concentration increases hydrophobicity, KH incorporation counteracts this effect by enhancing hydrophilicity. The strong statistical significance of these differences underscores the effectiveness of KH in modifying the hydrogel’s wettability.

Effective wound exudate absorption requires a hydrogel dressing with a high water absorption capacity. In this study, according to Figure 6c, all hydrogel formulations demonstrated excellent swelling properties, exceeding 500%. Notably, the 9GE_0.1KH hydrogel exhibited the highest swelling capacity (742.07 ± 89.61%), followed closely by 10GE_1.0KH (692.28 ± 46.40%). Furthermore, the statistical analysis revealed highly significant differences in swelling behavior among the hydrogel formulations. The most significant difference, as indicated by the **** (*p* < 0.0001) marker, was observed between 10GE and 9GE_0.1KH, where 9GE_0.1KH exhibited a significantly higher swelling ratio, indicating that 0.1% KH greatly enhanced water absorption. A moderately significant decrease (**, *p* < 0.01) was observed in 10GE compared to 9GE, suggesting that a higher gelatin concentration reduced swelling. Another significant difference (**, *p* < 0.01) was found between 10GE and 10GE_0.5KH, where 10GE_0.5KH had a higher swelling ratio, indicating that 0.5% KH improved water absorption compared to 10GE but to a lesser extent than 0.1% KH. This indicates that the incorporation of KH enhances the swelling properties of the hydrogels compared to their non-KH counterparts.

Figure 6d illustrates the biodegradation rates of the gelatin–KH hydrogels. Control hydrogels (9GE and 10GE) exhibited lower degradation rates (0.065 ± 0.017 mg/h and 0.085 ± 0.006 mg/h, respectively) compared to their KH-containing counterparts. The addition of KH increased degradation, with 9GE_0.1KH degrading at 0.079 ± 0.006 mg/h and 10GE_0.1KH at 0.090 ± 0.006 mg/h. Furthermore, the statistical analysis revealed significant differences in the biodegradation rates among the hydrogel formulations. The most significant difference (****, *p* < 0.0001) was observed between 9GE and 10GE_0.5KH, where 10GE_0.5KH exhibited a significantly higher biodegradation rate, indicating that 0.5% KH strongly enhanced hydrogel degradation. A highly significant increase (***, *p* < 0.001) was observed between 9GE and 10GE, suggesting that higher gelatin concentrations promoted biodegradability. Additionally, a significant difference (**, *p* < 0.01) was found between 9GE and 9GE_0.1KH, where 9GE_0.1KH exhibited a higher biodegradation rate, further supporting the role of KH in accelerating degradation.

These findings suggest that both an increased gelatin concentration and KH incorporation enhance biodegradation, with the most pronounced effect observed at 0.5% KH. However, despite these differences, all formulations maintained a degradation rate below 0.1 mg/h, ensuring controlled degradation over time.

### 3.4. Mechanical Strength of the Fabricated Hydrogels

**Figure 7:**
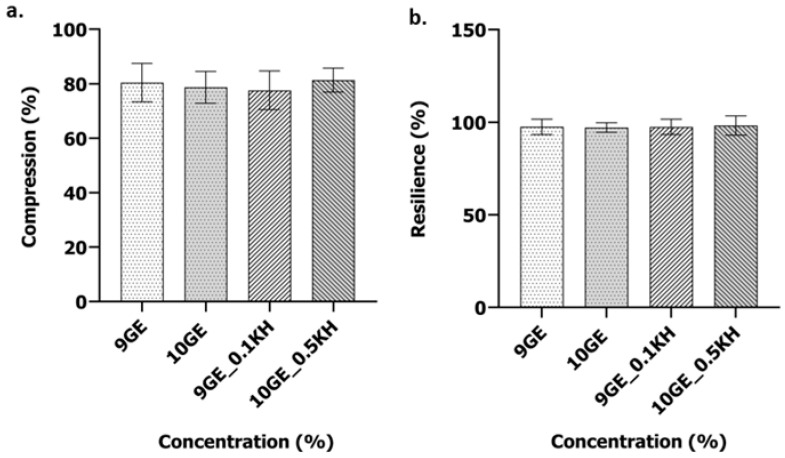
Analysis of the mechanical properties of 9GE, 10GE, 9GE_0.1KH and 10GE_10KH: (**a**) compression percentage and (**b**) percentage of resilience.

Figure 7a presents the compression analysis performed to assess the mechanical strength of the hydrogels, a key factor for successful implantation. No significant differences in compressive strength were observed between the scaffold groups. All scaffolds demonstrated the ability to support approximately 80% of a 300 g weight.

The resilience of the hydrogels, as shown in Figure 7b, defined as their capacity to regain their initial conformation following compressive deformation, was assessed. The statistical analysis revealed no significant intergroup differences in resilience. All hydrogels demonstrated complete shape recovery.

### 3.5. Chemical Characterization

**Figure 8:**
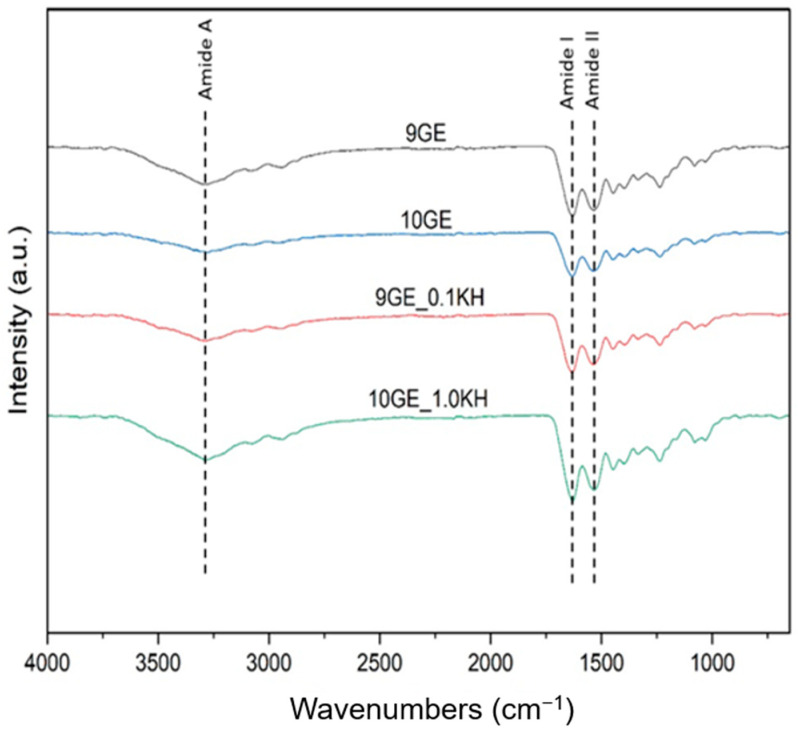
Chemical characterization of the hydrogels through FTIR spectra of the fabricated hydrogels.

FTIR spectroscopy provides a molecular fingerprint for identifying polymers, crosslinkers, and their chemical bonds. The spectra of the gelatin-based hydrogels reveal characteristic amide and hydroxyl regions, confirming their structural composition (Figure 8).

The broad absorption band at 3280–3745 cm^−1^ corresponds to O-H and N-H stretching (Amide A), indicating hydrogen bonding within the hydrogel matrix. The presence of Amide I (1625–1645 cm^−1^, C=O stretching) and Amide II (1525–1545 cm^−1^, N-H bending and C-N stretching) confirms the protein backbone of gelatin. Peaks within 1300–1370 cm^−1^ represent C-OH bending, characteristic of gelatin and genipin crosslinking, while C-H bending vibrations (1400–1450 cm^−1^) and C-O stretching (1080–1030 cm^−1^) further support the scaffold’s structure.

With the addition of Kelulut honey (KH), a shift in O-H stretching (~3289 cm^−1^) suggests enhanced hydrogen bonding between honey and gelatin. The emergence of peaks at 2800–2950 cm^−1^ (C-H stretching) confirms the presence of sugars from honey, while peaks at 875–699 cm^−1^ (C-O and C-H vibrations) indicate further sugar-related interactions. Additionally, new absorption bands at 2166–2212 cm^−1^ suggest the presence of C≡C or C≡N stretching, likely from bioactive compounds in honey.

### 3.6. 3D-Microporous Structure Hydrogel

**Figure 9:**
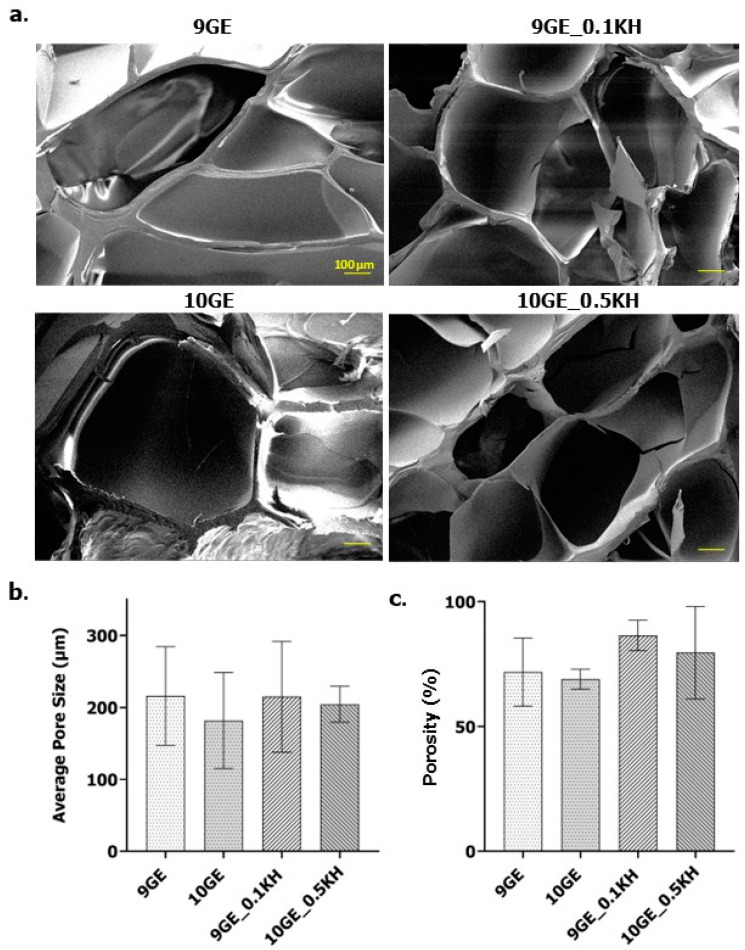
(**a**) SEM images showing the cross-sectional microporous structure of the hydrogels at 100× magnification; (**b**) average pore size (μm); and (**c**) porosity percentage.

The SEM micrographs of gelatin-based hydrogels, with and without Kelulut honey (KH) addition, revealed heterogenic porous structures (Figure 9a). The non-Kelulut honey-infused hydrogels, 9GE and 10GE, exhibited more compact microstructures with fewer and larger pores compared to the Kelulut honey variants. In contrast, the crosslinked hydrogels, especially with KH addition, showed more pronounced porosity and higher interconnectivity. 9GE_0.1KH displayed a rougher, irregular pore structure, while 10GE_0.5KH demonstrated a highly porous and interconnected network. All images were analyzed using ImageJ software (version 1.54k, NIH, Bethesda, MD, USA)showing that KH enhances porosity and interconnectivity in the crosslinked hydrogels. After the freeze-drying process, it was found that 10GE had the highest average pore size of 241.793 ± 39.327 μm followed by 9GE with an average pore size of 226.174 ± 50.915 μm, while 9GE_0.1KH and 10GE_0.5KH had 186.784 ± 20.936 μm and 206.292 ± 17.615 μm average pore size, respectively, as can be seen in Figure 9b.

The addition of Kelulut honey both at 0.1KH and 0.5KH correspondingly increased the porosity of the hydrogels, which could be attributed to the interaction between honey and the gelatin–genipin network, enhancing the scaffold’s ability to form a more porous structure. The percentage of the hydrogel’s porosity can be found in Figure 9c. The microstructure of these hydrogels shows a promising resemblance to natural tissue, especially with higher KH concentrations.

The EDX analysis revealed the elemental composition of gelatin–genipin hydrogels with and without Kelulut honey, which can be seen in Table 1. Both 9GE and 10GE showed similar carbon (56.07 ± 0.23 and 56.70 ± 1.07%), oxygen (28.7 ± 1.11 and 28.2 ± 1.90%), and nitrogen (15.2 ± 0.86 and 15.1 ± 1.72%) levels. Adding 0.1% Kelulut honey to 9GE significantly increased the carbon (60.3 ± 4.99%) and oxygen (30.6 ± 1.50%) levels while decreasing the nitrogen level (13.7 ± 0.65%), consistent with honey’s carbohydrate content. Similarly, 0.5% honey in 10GE increased the oxygen level (29.6 ± 0.19), with a less pronounced change in the carbon level (54.6 ± 0.52%) and a slight increase in the nitrogen level (15.7 ± 0.57%). These changes confirm honey incorporation and suggest differing interactions based on the gelatin and honey concentrations.

**Figure 10:**
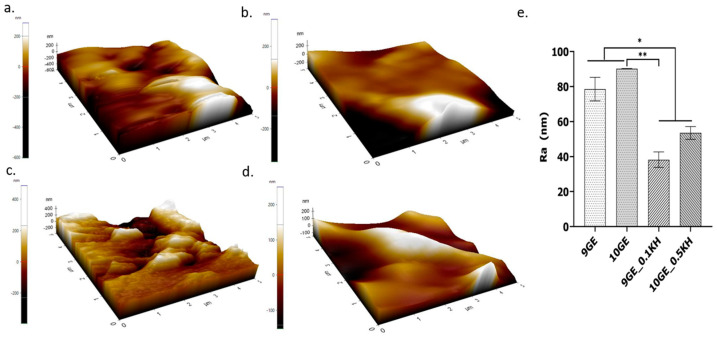
AFM image analysis of the surface roughness: (**a**) 9GE, (**b**) 9GE_0.1KH, (**c**) 10GE, (**d**) 10GE_0.5KH. (**e**) Graph of the Ra values (** indicates *p* < 0.01 and * indicates *p* < 0.05).

Figure 10 shows the surface topology of the hydrogel samples based on AFM scanned images, including the mean surface roughness for all samples. The surface roughness of the hydrogel samples will affect cell adhesion and cell behavior. Overall, Figure 10a,c, which represent gelatin and genipin-crosslinked hydrogels without the addition of Kelulut honey (KH), exhibit a rougher surface topology with distinct peaks, valleys, and significant height variations, indicating a rougher surface. Among them, 10GE has the roughest surface topology and highest mean surface roughness, while 9GE_0.1KH has the smoothest surface topology and mean surface roughness.

Figure 10e shows the roughness average (Ra) value, with 10GE having the highest Ra value of 90.279 ± 0.113 nm followed by 9GE (73.827 ± 6.271 nm) and 10GE_0.5KH (56.078 ± 3.631 nm), and 9GE_0.1KH (41.369 ± 4.413 nm) having the lowest Ra value and being the smoothest hydrogel sample. A moderately significant difference (**, *p* < 0.01) was observed between 10GE and 9GE_0.1KH, confirming that 9GE_0.1KH exhibited a significantly lower roughness, emphasizing the smoothing effect of KH. Additionally, a lower level of significance (*, *p* < 0.05) was found in three comparisons, 9GE_and 9GE_0.1KH, 9GE and 10GE_0.5KH, and 10GE and 10GE_0.5KH, further indicating that KH incorporation plays a role in modifying the surface characteristics. As the percentage of gelatin is increased, the mean surface roughness increases, while the addition of Kelulut honey will reduce the mean surface roughness and make the hydrogel samples smoother.

## 4. Discussion

The findings present the impacts of Kelulut honey addition and genipin crosslinking on the physicochemical and mechanical properties of the resulting hydrogels for all four hydrogel samples. All four groups presented different impacts on polymerization behavior, physicochemical properties, tensile strength, and chemical composition. The dose–response study showed that fibroblast viability varied across the samples, with Kelulut honey (KH) addition influencing cellular responses. Differences in the polymerization time across the groups showed that KH played a role in gelation. Further analysis of the hydrogels’ physicochemical and tensile properties showed that KH influenced their swelling behavior, stability, and structure. The chemical analysis confirmed crosslinking success, with microporosity measurements providing insights into the hydrogels’ potential for influencing cellular behavior. These findings provide a foundation for further discussion on the impacts of the addition and crosslinking with genipin, in this case, their potential for cutaneous tissue loss. This section will discuss these findings in more detail with respect to their relevance and broader value.

### 4.1. Kelulut Honey’s Effect on Human Dermal Fibroblasts (HDFs)

The findings of this study provide significant insights into the dose-dependent effects of Kelulut honey on fibroblast viability over time. The highest viability was observed at a 0.1% concentration, particularly at 24 h, which aligns with previous studies indicating that certain natural honey components, such as flavonoids and phenolic compounds, can promote cell proliferation by providing antioxidant and anti-inflammatory benefits [34,46]. However, at higher concentrations (4.0% and 10.0%), a significant reduction in cell viability was observed. At 4.0%, the decline in cell viability was noticeable, particularly at the 72-h time point, suggesting that prolonged exposure to this concentration exerts cytotoxic effects. The cytotoxicity became even more pronounced at 10.0%, where fibroblast viability was nearly eliminated across all time points. These findings align with prior research demonstrating that high concentrations of honey, particularly those with strong osmotic potential and a high sugar content, can lead to hyperosmotic stress, ultimately causing cell death [47].

The observed cytotoxicity for higher concentrations of Kelulut honey may be attributed to several factors. One possible mechanism involves oxidative stress, where high concentrations of Kelulut honey led to the excessive production of reactive oxygen species (ROS) [48]. While low concentrations of Kelulut honey may act as antioxidants, mitigating oxidative damage, higher concentrations may paradoxically induce oxidative stress, triggering apoptosis or necrosis in fibroblasts. This oxidative imbalance could be due to the presence of phenolic compounds, which, at high levels, can act as pro-oxidants rather than antioxidants. Additionally, the hyperosmotic nature of honey at elevated concentrations may cause the dehydration of cells, leading to a loss of membrane integrity and subsequent cell death [49].

The trend of decreasing viability over time across all concentrations confirms that prolonged exposure to even moderately high concentrations of Kelulut honey can exacerbate cytotoxic effects. This cumulative impact might be attributed to the gradual accumulation of ROS or other cytotoxic metabolites present in honey [48]. Additionally, the metabolic burden imposed by honey’s high sugar content may interfere with normal cellular energy homeostasis, further compromising fibroblast survival. While studies on alginate bioinks, like Datta et al., 2018, show beneficial effects of honey at 1–5%, prolonged exposure to even slightly higher concentrations, as seen in this study, raises concerns about long-term stability and biocompatibility [50].

### 4.2. Successful Fabriction and Crosslinking

Advancements in tissue engineering for wound healing are crucial to address the limitations of current treatments and achieve functional skin regeneration. These advancements focus on developing biomaterial inks that enhance biocompatibility, mimic the natural extracellular matrix, and maintain cell viability during the bioprinting process. Specifically, these inks must possess appropriate mechanical properties for withstanding physiological stress, facilitate vascularization and skin appendage regeneration for full functionality, and exhibit ease of printing for precise fabrication of complex skin structures [51]. To this end, this research aims to design an optimal, polymerizing, and non-toxic injectable hydrogel suitable for cell therapies and as a potential biomaterial ink for future 3D bioprinting.

This study successfully formulated hydrogels by combining natural polymers, gelatin, and Kelulut honey across several formulations, demonstrating potential therapeutic efficacy for chronic skin wounds. Genipin was incorporated to enhance the hydrogel’s structural integrity and promote wound healing via the stimulation of cellular proliferation [52]. Optimal formulations, specifically 9% GE with 0.1% Kelulut honey (9GE_0.1KH) and 10% GE with 0.5% Kelulut honey (10GE_0.5KH), were identified, exhibiting a three-minute polymerization time at room temperature. This controlled polymerization rate allows for an adequate clinical application time before gelation [29], while also preventing premature solidification that could hinder extrusion during 3D bioprinting. Genipin’s presence facilitates this through the formation of covalent crosslinks with gelatin’s amino-polymeric constituents [53].

One pathway involves nucleophilic attack by the free amino groups in gelatin on the C3 carbon of genipin, triggering the opening of the dihydropyran ring in the genipin structure [53]. This reaction leads to the formation of an interconnected gelatin network, reinforcing the hydrogel’s mechanical strength and stability. In the second pathway, the amino groups in gelatin react with the genipin carboxyl group, forming amide bonds [53]. These covalent amide linkages act like molecular zippers, strengthening the hydrogel and improving its resistance to degradation. Together, these mechanisms create a robust, crosslinked network that enhances the hydrogel’s mechanical properties and durability. Beyond these primary crosslinking mechanisms, oxygen radical-induced polymerization of genipin may also occur, resulting in further stabilization of the hydrogel network. This additional reaction contributes to the characteristic bluish hue observed in genipin-crosslinked hydrogels, which serves as a visual indicator of successful crosslinking [53]. The controlled formation of this crosslinked structure ensures that the hydrogel remains structurally stable while maintaining sufficient elasticity for biomedical applications.

### 4.3. Physicochemical Properties and Wound Healing

The performance of an ideal hydrogel was also evaluated by examining its physicochemical characteristics. Unlike healthy skin, injured skin tends to lose a considerable amount of water and moisture. The swelling ratio of the fabricated gelatin–genipin-crosslinked hydrogels, both with and without Kelulut honey (KH), demonstrated an acceptable level that enables them to effectively absorb excess wound exudate at injury sites. All four hydrogels exhibited a swelling ratio exceeding 500%, especially with the addition of KH, making them ideal candidates for wound healing applications. Gelatin, a primary component, inherently exhibits hydrophilic characteristics due to the presence of polar amino acid residues such as those containing hydroxyl (-OH), carboxyl (-COOH), and amine (-NH2) groups [51]. These functional groups readily engage in hydrogen bonding with water molecules, facilitating the initial uptake of fluid into the gelatin–genipin hydrogel matrix. 9GE_0.1KH showed the highest swelling capacity (742.07 ± 89.61%), significantly outperforming 10GE (692.28 ± 46.40%), as indicated by the **** (*p* < 0.0001) significance marker. This highlights that the addition of Kelulut honey at a low concentration (0.1%) significantly enhances the swelling capacity of the hydrogel. With a 500% water retention capacity, these hydrogels can prevent the buildup of exudates in the wound area while effectively absorbing water [54,55,56]. These results are influenced by and correlate with the hydrophilic properties of both gelatin and Kelulut honey. Kelulut honey possesses hydrophilic characteristics due to its hygroscopic nature attributed to its high sugar content, which enables it to absorb moisture from the surrounding environment [57]. These sugars are characterized by the presence of numerous hydroxyl (-OH) groups, which are highly effective at forming hydrogen bonds with water molecules, thereby enhancing the overall water absorption capacity of the hydrogel [58]. Additionally, Kelulut honey contains phenolic compounds and flavonoids, which also possess polar functional groups that contribute to their hydrophilic nature [58]. Furthermore, the retained water contributes to maintaining a moist wound environment, which is widely recognized as essential for facilitating cellular activities such as cell migration, proliferation, and the deposition of new extracellular matrix components, all critical for successful tissue repair.

The increase in Kelulut honey concentration within the composite hydrogel scaffold enhances the ratio of hydrophilic to hydrophobic groups, as well as water retention, which leads to scaffolds that are more flexible and capable of withstanding mechanical stress [59]. The contact angle measurements provide additional evidence of how KH incorporation improves the hydrophilicity of the hydrogel. A significant decrease in the contact angle was observed upon the addition of KH. Specifically, 9GE_0.1KH and 10GE_0.5KH demonstrated reduced contact angles, which were statistically significant **** (*p* < 0.0001), indicating enhanced wettability and thus a better interaction with fluids. The moderately significant difference (**, *p* < 0.01) between 9GE and 9GE_0.1KH further supports the claim that KH enhances the hydrophilic properties of the hydrogels, which is beneficial for maintaining an optimal moisture balance at the wound site [60]. Notably, 10GE exhibited a more hydrophobic nature compared to 9GE_0.1KH, as shown by the higher contact angle (***, *p* < 0.001), reinforcing the idea that the inclusion of KH overcomes the inherent hydrophobicity of higher gelatin concentrations and improves fluid absorption, which is necessary for wound healing. Kelulut honey contains various hydrophilic compounds, including sugars like trehalulose, aliphatic organic acids, short-chain fatty acids, flavonoids, phenolic acids, and polyphenols [58]. The hydroxyl groups present in sugars and the polar functional groups in phenolic compounds and flavonoids can readily interact with water molecules at the hydrogel’s surface through hydrogen bonding [61]. This interaction increases the surface energy of the hydrogel, making it more attractive to water and thus improving its wettability, which is reflected in a reduced contact angle. The incorporation of these hydrophilic compounds likely alters the surface chemistry of the hydrogel, increasing the density of water-attracting functional groups. This highlights the potential of Kelulut honey to modulate the surface properties of gelatin-based hydrogels, making them more suitable for interacting with the aqueous environment of a wound.

Furthermore, hydrogels need to have an adequate WVTR to maintain the proper moisture level in the wound area. WVTR characterization is a crucial factor for wound healing applications to ensure the wound stays moist. The ideal WVTR level for a potential skin substitute should be above 1500 g/m^2^/h, as this helps keep the wound hydrated and prevents excessive dehydration. Hence, all the fabricated gelatin–genipin-crosslinked hydrogels both with and without Kelulut honey have good WVTRs, as the results are within the hydrogel range and allow for a good moisture balance [62]. This suggests that within the tested ranges of concentrations of both Kelulut honey and gelatin, the variations in the crosslinking density might not have significantly altered the overall water vapor permeability, even though they could have affected the pore morphology.

In addition, another crucial factor to be tested and considered is the in vitro biodegradation of the hydrogel formulation, as a fast biodegradation rate of the biomaterials after implantation on the wound site is the current existing problem with other hydrogel formulations. Figure 6d shows the results of the rate of biodegradation of the hydrogels. The crosslinked gelatin–genipin hydrogels, which are the control hydrogels, showed prolonged durability and lower degradation rates compared to hydrogels with Kelulut honey, signaling that the addition of Kelulut honey increases the biodegradation rate while still maintaining a degradation rate below 0.1 mg/h, which is within the acceptable range of degradation. The selected hydrogel for wound healing applications should remain intact for a minimum of 14 days before fully degrading at the implantation site, which should have a maximum degradation rate of 0.2 mg/h. The experimental results show that gelatin–genipin-crosslinked hydrogels without Kelulut honey demonstrated extended durability and slower degradation rates. This is likely due to the genipin-crosslinked network providing resistance to enzymatic breakdown. However, incorporating Kelulut honey led to a higher biodegradation rate, though it remained within an acceptable range (below 0.1 mg/h). This acceleration in degradation may be linked to several possible mechanisms, including the presence of various enzymes in Kelulut honey, such as diastase and invertase [30,63].

### 4.4. Mechanical Properties and Their Significance

The mechanical properties of the hydrogels, which are essential for wound healing applications, were assessed via compression and resilience testing (Figure 7). All formulations exhibited statistically similar compression percentages (Figure 7a), indicating that Kelulut honey addition did not substantially alter compressibility. Resilience, a measure of the hydrogel’s capacity to recover its initial form following deformation, is essentially linked to the polymer network’s crosslinking density and the mobility of the polymer chains within that network. The general consistency in resilience across the formulations implies that Kelulut honey, even at a higher 0.5% concentration, does not induce substantial alterations in the hydrogel’s elastic behavior. While minor variations in resilience were observed (Figure 7b), particularly a slight decrease with 0.1% Kelulut honey (9GE_0.1KH), these differences were not considered significant. These results suggest that the incorporation of Kelulut honey, within the tested concentrations, does not critically impact the overall mechanical behavior of the hydrogels, maintaining their suitability for supporting tissue regeneration.

The observed maintenance of consistent mechanical behavior across the hydrogel formulations, despite the inclusion of Kelulut honey, is a crucial factor for their potential application in wound healing, as it important to ensure that the bioactive compounds in Kelulut honey do not affect the hydrogel’s overall polymeric network. These properties are essential for providing structural support to regenerating tissues and facilitating optimal cell migration and proliferation [64]. The preservation of these mechanical attributes underscores the hydrogel’s suitability as a mechanically robust and biocompatible scaffold for tissue regeneration. It is important to note that the mechanical properties of hydrogels are essential for their application in wound healing, because the mechanical properties of the hydrogel need to be similar to the elasticity and flexibility of the tissues that the hydrogel is replacing [64].

### 4.5. Chemical and Structural Characterization

FTIR spectroscopy provides insights into the molecular structure of the gelatin-based hydrogels, revealing key functional groups associated with gelatin, genipin crosslinking, and interactions with Kelulut honey (KH) (Figure 8). The broad absorption band between 3280 and 3745 cm^−1^ corresponds to O-H and N-H stretching (Amide A), indicating hydrogen bonding within the hydrogel matrix, which contributes to its stability and mechanical properties.

Peaks at 1625–1645 cm^−1^ (Amide I, C=O stretching) and 1525–1545 cm^−1^ (Amide II, N-H bending and C-N stretching) confirm the presence of gelatin, while peaks between 1300 and 1370 cm^−1^ (C-OH bending) reflect the crosslinking of gelatin with genipin. Additional C-H bending (1400–1450 cm^−1^) and C-O stretching (1080–1030 cm^−1^) further validate the hydrogel’s structure [65].

The addition of KH shifts the O-H stretching band to ~3289 cm^−1^, indicating enhanced hydrogen bonding between honey and gelatin, which may improve the hydrogel’s stability. New peaks in the 2800–2950 cm^−1^ region (C-H stretching) confirm the presence of sugars from the honey, while peaks at 875–699 cm^−1^ (C-O and C-H vibrations) suggest interactions between the honey’s sugars and the gelatin matrix. Furthermore, bands at 2166–2212 cm^−1^, attributed to C≡C or C≡N stretching, likely indicate bioactive honey compounds contributing to the hydrogel’s functional properties.

An EDX analysis was performed to assess the elemental composition of gelatin–genipin hydrogels with and without Kelulut honey (KH). The 9GE and 10GE samples showed similar elemental contents of approximately 56% carbon, 28% oxygen, and 15% nitrogen, reflecting the gelatin network structure [66]. Adding 0.1% KH to 9GE significantly increased the carbon (60.3 ± 4.99%) and oxygen (30.6 ± 1.50%) contents while decreasing the nitrogen content (13.7 ± 0.65%), consistent with the high carbohydrate content of honey. This suggests that Kelulut honey’s sugars were incorporated into the hydrogel matrix, replacing some of the nitrogenous gelatin components. In contrast, in 10GE_0.5KH, the carbon content showed a smaller change (54.6 ± 0.52%), but the oxygen content increased (29.6 ± 0.19%) and the nitrogen content was slightly increased (15.7 ± 0.57%). This indicates a saturation effect where a higher concentration of Kelulut honey resulted in a more subtle incorporation of sugars, with a modest increase in oxygen levels, likely from bioactive compounds in Kelulut honey and possibly due to a shift in the interaction mechanism [65].

The concentration-dependent nature of KH incorporation suggests that lower concentrations favor direct sugar–gelatin interactions, while higher concentrations may involve additional intermolecular forces or phase separation effects. The greater incorporation of oxygen in both cases supports the presence of hydroxyl-rich sugar molecules within the hydrogel. The smaller change in the carbon content at higher KH concentrations may indicate a shift in how honey integrates into the network, possibly through more complex hydrogen bonding or even minor phase separation.

Furthermore, the observed nitrogen reduction in 9GE_0.1KH suggests that gelatin’s proteinaceous regions are partially replaced by Kelulut honey sugars, altering the hydrogel’s molecular composition. The slight increase in the nitrogen content in 10GE_0.5KH may be due to the presence of nitrogen-containing bioactive compounds from KH, which could enhance the hydrogel’s biological activity. These shifts in elemental composition provide valuable insights into the material’s chemical modifications, which could influence its mechanical properties, degradation rate, and potential biomedical applications.

These results confirm that Kelulut honey is incorporated into the hydrogels, with varying interactions depending on the honey concentration, which could influence the hydrogel’s properties for biomedical applications.

### 4.6. Microporous Structure and 3D Bioprinting

As the goal is to characterize the formulation of the injectable hydrogel as a potential bioink for 3D bioprinting, the initial data were gathered by assessing the overall appearance, as well as evaluating the average pore sizes through an analysis of the SEM micrographs shown in Figure 8. The porosity of the hydrogels was also tested through the water displacement method and the surface roughness of the hydrogels was analyzed through AFM images to estimate the porosity and surface roughness of the hydrogels once printed.

The SEM micrographs of gelatin-based hydrogels, with and without Kelulut honey (KH), reveal distinct differences in their microstructure. The non-KH hydrogels (9GE and 10GE) showed a more compact structure with fewer, larger pores, suggesting a denser matrix due to stronger gelatin–genipin interactions [67]. As gelatin and genipin interact and form covalent bonds, they reinforce the hydrogel network, resulting in a denser matrix and more compact structure [54]. The concentration of gelatin in the hydrogel matrix directly affects the density of these functional groups, with higher concentrations (10GE) providing a greater number of potential interaction sites compared to lower concentrations (9GE), making the hydrogel even denser and compact, as revealed by the SEM data. In contrast, the hydrogels with KH exhibited increased porosity and interconnectivity. This enhancement is likely due to KH interacting with the gelatin, either by introducing additional crosslinking or reducing rigidity, which facilitates the formation of smaller, interconnected pores. As KH contains high percentage of sugars, especially maltose and fructose, it alters the hydrogen-bonding structure of nearby water molecules in the gelatin–genipin matrix, leading to a less dense network with smaller pores [58]. Additionally, KH’s reducing sugars, such as maltose, fructose, and glucose, can undergo the Maillard reaction with gelatin’s free amine groups, competing with genipin for binding sites and altering the overall crosslinking density [58,68]. The 9GE_0.1KH sample displayed a rougher, irregular pore structure, while the 10GE_0.5KH variant showed a highly porous and interconnected network [67]. The ImageJ (version 1.54k, NIH, Bethesda, MD, USA) analysis confirmed that KH addition consistently increased both porosity and interconnectivity. This aligns with findings from similar studies, where natural additives like Kelulut honey alter the hydrogel’s pore structures by acting as plasticizers, reducing the overall gelatin–genipin matrix rigidity [69].

After freeze-drying, the average pore size was largest in 10GE (241.793 ± 39.327 μm) and 9GE (226.174 ± 50.915 μm), while 9GE_0.1KH and 10GE_0.5KH had smaller pore sizes (186.784 ± 20.936 μm and 206.292 ± 17.615 μm, respectively), indicating that KH reduces the pore size while increasing the overall porosity. This can be attributed to more efficient crosslinking and the formation of a lesser dense honey–gelatin network [70]. Another factor would be Kelulut honey’s high water content, which could lead to the dilution of the gelatin concentration within the hydrogel, potentially hindering the efficient formation of a dense crosslinked network and contributing to a reduced average pore size [71].

The increased porosity in KH-infused hydrogels is likely due to the honey’s ability to disrupt the gelatin network, creating a more porous structure. The components of Kelulut honey, particularly the organic acids that lower the pH, can significantly influence the effectiveness of genipin crosslinking [71]. While genipin can crosslink gelatin under slightly acidic conditions, lower pH values can hinder the reaction. The acidic nature of KH might slow down or reduce the extent of genipin crosslinking in KH-containing hydrogels. This could result in a less dense crosslinked network compared to non-KH hydrogels at the same gelatin concentration, potentially explaining the higher porosity and interconnectivity observed in the KH-containing hydrogels. This is significant for biomedical applications, where porosity influences cell infiltration and tissue regeneration. Hydrogels with higher porosity and interconnected pores better resemble natural tissues, making them suitable for tissue engineering, as they mimic the porous structure of native extracellular matrices and are particularly advantageous for regenerative medicine because they provide an optimal environment for cell attachment, migration, and differentiation [72].

As the gelatin concentration increases, the mean surface roughness tends to rise due to the formation of a denser, more rigid polymer network. However, the addition of Kelulut honey (KH) consistently reduces the surface roughness, resulting in smoother hydrogels. This can be attributed back to the plasticizing effect of KH, which could reduce the rigidity of the gelatin network and facilitate the formation of a smoother surface [69]. In addition, honey’s inherent viscosity and its capacity to form a cohesive layer on surfaces can assist in smoothing out the surface roughness by filling micro-voids and producing a more consistent surface layer [69].

In conclusion, the data indicate that increasing the gelatin concentration increases the surface roughness due to better crosslinking with genipin and a denser network, while Kelulut honey reduces the roughness as it alters the structural network of the hydrogel, contributing to smoother hydrogel surfaces more conducive to cell adhesion and behavior [67].

### 4.7. Limitations and Future Directions

The current study primarily focuses on in vitro analyses of the hydrogels’ properties and their impacts on cell viability. While promising results were obtained, these findings may not fully reflect the behavior of the hydrogel in a living organism [19]. The in vivo application of these hydrogels remains unexplored, and assessing their long-term safety and effectiveness in an animal model or clinical trials is critical. Additionally, the study utilized primary human dermal fibroblasts (HDFs) for assessing KH’s cytotoxicity and effects on cell growth. However, the use of other cell types, such as keratinocytes or endothelial cells, could provide a more comprehensive understanding of KH’s performance in wound healing, particularly regarding epithelialization and vascularization.

Moreover, while the study evaluated basic physicochemical properties such as the swelling ratio, biodegradation, and contact angle, it did not incorporate external factors like infection or the presence of various inflammatory mediators, which could impact hydrogel functionality in a wound environment. The biodegradation of the hydrogels was tested over a short period, and a longer-term evaluation is required to understand the full degradation timeline. Premature degradation or persistence in the wound site could affect the healing process and tissue regeneration. Lastly, the study explored the effects of genipin crosslinking at a single concentration. It would be beneficial to assess the impact of varying genipin concentrations on the mechanical properties and cytotoxicity of the hydrogels, as this could offer insights into optimizing the formulation for clinical use.

As for the future directions, it will be beneficial to conduct in vivo studies to evaluate the performance of the gelatin–Kelulut honey hydrogels in animal models [72,73]. These studies will help determine the biocompatibility, wound healing efficacy, and long-term stability of the hydrogels in real biological systems, particularly for chronic wound treatment. Upon obtaining successful preclinical data, clinical trials involving human participants should be conducted. These clinical trials would provide critical insights into the hydrogel’s safety, efficacy, and practicality in real-world wound care and allow for further optimization [71].

Additionally, future research can expand on the combination of gelatin with other bioactive materials, such as chitosan, alginate, or silk fibroin. These materials could further enhance the hydrogel’s mechanical strength, biocompatibility, and wound healing properties [14]. Another promising direction is the development of “smart” hydrogels that can respond to changes in the wound environment, such as variations in pH, temperature, or enzyme activity. Incorporating responsive elements into the hydrogel could allow for the controlled release of therapeutic agents like growth factors, antibiotics, or anti-inflammatory compounds, thereby improving wound healing.

## 5. Conclusions

In conclusion, the development of gelatin-based hydrogels crosslinked with genipin and incorporating Kelulut honey has shown promising results for wound healing applications. The study demonstrated that these hydrogels possess favorable physicochemical properties, including an excellent swelling capacity, biodegradation rates, and hydrophilicity, making them suitable for chronic wound care. Furthermore, the addition of Kelulut honey provides enhanced antimicrobial and anti-inflammatory effects, which are crucial for promoting tissue regeneration and reducing the risk of infection in wound sites.

Despite the encouraging in vitro findings, further research is required to assess the hydrogels’ performance in vivo, particularly regarding their long-term biocompatibility, biodegradation, and effectiveness in real-world clinical scenarios. The integration of 3D bioprinting and the incorporation of additional bioactive materials could further improve the functionality and customization of these hydrogels, offering a more tailored solution for individual patients. Moreover, future studies exploring the combination of hydrogels with stem cells, growth factors, and other therapeutic agents may accelerate the healing process and improve the overall clinical outcomes.

## Figures and Tables

**Table 1:** Elemental analysis of hydrogels with EDX. All hydrogels possessed different elemental compositions, including oxygen, carbon, and nitrogen.

Sample	C (%)	O (%)	N (%)
9GE	56.07 ± 0.23	28.7 ± 1.11	15.2 ± 0.86
9GE_0.1KH	60.3 ± 4.99	30.6 ± 1.50	13.7 ± 0.65
10GE	56.7 ± 1.07	28.2 ± 1.90	15.1 ± 1.72
10GE_0.5KH	54.6 ± 0.52	29.6 ± 0.19	15.7 ± 0.57

## Data Availability

The original contributions presented in this study are included in the article. Further inquiries can be directed to the corresponding author.

## References

[1] Loh E.Y.X., Mohamad N., Fauzi M.B., Ng M.H., Ng S.F., Amin M.C.I.M. (2018). Development of a bacterial cellulose-based hydrogel cell carrier containing keratinocytes and fibroblasts for full-thickness wound healing. Sci. Rep..

[2] Han S.-K. (2023). Basics of Wound Healing. Innovations and Advances in Wound Healing.

[3] Zawani M., Fauzi M.B. (2021). Injectable Hydrogels for Chronic Skin Wound Management: A Concise Review. Biomedicines.

[4] Hoang T.P.N., Ghori M.U., Ousey K.J., Conway B.R. (2022). Current and advanced therapies for chronic wound infection: An overview of chronic wounds, including their physiology, causes and management options. Pharm. J..

[5] Kamolz L.P., Lumenta D.B., Kitzinger H.B., Frey M. (2008). Tissue engineering for cutaneous wounds: An overview of current standards and possibilities. European Surgery. Eur. Surg..

[6] Ples M., Glik J., Misiuga M., Skotnicka J., Kawecki M., Nowak M. (2016). Chronic wounds and their treatment. Skin substitutes and allogeneic transplantations. J. Orthop. Trauma Surg. Relat. Res..

[7] Saeed S., Martins-Green M. (2024). Assessing Animal Models to Study Impaired and Chronic Wounds. Int. J. Mol. Sci..

[8] Sizemore B., Singh K., Sen C.K. (2022). A Sociogenomic Analysis of Chronic Wounds. Proc. IMPRS.

[9] Lin C., Ailing H., Caifei L.B., Yuan L. (2024). Impact of Symptoms on Quality of Life in Patients with Chronic Wounds. Adv. Ski. Wound Care.

[10] Saifullah Q., Sharma A. (2024). Current Trends on Innovative Technologies in Topical Wound Care for Advanced Healing and Management. Curr. Drug Res. Rev..

[11] Devdikar S., Reza A., Parsania T., Jha N., Vardey M., Guntupalli A. (2024). Analysis of Efficacy of Collagen Dressing Versus Conventional Dressing in Chronic Wound. Int. J. Adv. Res..

[12] Kulprachakarn K., Nantakool S., Rojawat C., Ounjaijean S., Pongtam S., Prasannarong M., Rerkasem K. (2021). Effectiveness of combined conventional treatment with a tailored exercise training program on wound healing in patients with venous leg ulcer: A randomized controlled trial. J. Tissue Viability.

[13] Colin V., Listiana D. (2022). Efektivitas Perawatan Luka Dengan Metode Perawatan Luka Modern Dan Perawatan Luka Konvensional Pada Pasien Diabetes Melitus. Care J. Ilm. Ilmu Kesehat..

[14] Mahajan N., Soker S., Murphy S.V. (2024). Regenerative Medicine Approaches for Skin Wound Healing: From Allografts to Engineered Skin Substitutes. Curr. Transplant. Rep..

[15] Abuhamad A.Y., Masri S., Fadilah N.I.M., Alamassi M.N., Maarof M., Fauzi M.B. (2024). Application of 3D-Printed Bioinks in Chronic Wound Healing: A Scoping Review. Polymers.

[16] Kim Y.S., Shin Y.S. (2024). Surface Functionalization of 3D-Printed Bio-Inspired Scaffolds for Biomedical Applications: A Review. Biomimetics.

[17] Mandal A., Chatterjee K. (2024). The 3D/4D Printing of Polymeric Scaffolds for Bone Tissue Engineering. Emerging Materials and Technologies for Bone Repair and Regeneration.

[18] Zhou X., Yu X., You T., Zhao B., Dong L., Huang C., Zhou X., Xing M., Qian W., Luo G. (2024). 3D Printing-Based Hydrogel Dressings for Wound Healing. Adv. Sci..

[19] Wang Y., Yuan X., Yao B., Zhu S., Zhu P., Huang S. (2022). Tailoring bioinks of extrusion-based bioprinting for cutaneous wound healing. Bioact. Mater..

[20] Yuan X., Zhu W., Yang Z., He N., Chen F., Han X., Zhou K. (2024). Recent Advances in 3D Printing of Smart Scaffolds for Bone Tissue Engineering and Regeneration. Adv. Mater..

[21] Zhai X., Wu Y., Tan H. (2023). Gelatin-based Targeted Delivery Systems for Tissue Engineering. Curr. Drug Targets.

[22] Kapoor D., Verma K., Jain S., Sharma S. (2024). Gelatin-based Hydrogels for Drug Delivery: A Recent Update. Biomaterial-Based Hydrogels.

[23] Wei Z., Zuo Y., Wu E., Huang L., Qian Y., Wang J., Chen Z. (2024). Highly biocompatible, antioxidant and antibacterial gelatin methacrylate/alginate—Tannin hydrogels for wound healing. Int. J. Biol. Macromol..

[24] Wahba M.I. (2024). A comprehensive review on genipin: An efficient natural cross-linker for biopolymers. Polym. Bull..

[25] Ahmed R., Hira N.U.A., Wang M., Iqbal S., Yi J., Hemar Y. (2023). Genipin, a natural blue colorant precursor: Source, extraction, properties, and applications. Food Chem..

[26] Jovanović M., Petrović M., Stojanović D., Radulović N., Pantelić D., Stajčić I., Uskoković P. (2024). 3D-Printed Gelatin-Based Scaffold crosslinked by GenIPin: Evaluation of mechanical properties and biological effect. Biopolymers.

[27] Scomazzon L., Ledouble C., Dubus M., Braux J., Guillaume C., Bouland N., Baldit A., Boulmedais F., Gribova V., Mauprivez C. (2024). An increase in Wharton’s jelly membrane osteocompatibility by a genipin-cross-link. Int. J. Biol. Macromol..

[28] Masri S., Maarof M., Mohd N.F., Hiraoka Y., Tabata Y., Fauzi M.B. (2022). Injectable Crosslinked Genipin Hybrid Gelatin–PVA Hydrogels for Future Use as Bioinks in Expediting Cutaneous Healing Capacity: Physicochemical Characterisation and Cytotoxicity Evaluation. Biomedicines.

[29] Nike D.U., Katas H., Mohd N.F., Hiraoka Y., Tabata Y., Idrus R.B.H., Fauzi M.B. (2021). Characterisation of Rapid In Situ Forming Gelipin Hydrogel for Future Use in Irregular Deep Cutaneous Wound Healing. Polymers.

[30] Mustafa M.Z., Vit P. (2024). Honeyomics and Industrialisation of Madu Kelulut as a Health Supplement: Are We Ready for Scale-Up?. Malays. J. Med Sci..

[31] Rosli M.A., Nasir N.A.M., Mustafa M.Z., Othman M.A., Zakaria Z., Halim A.S. (2024). Effectiveness of stingless bee (Kelulut) honey versus conventional gel dressing in diabetic wound bed preparation: A randomized controlled trial. J. Taibah Univ. Med Sci..

[32] Khairan K., Mudatsir M., Diah M., Rizal S., Putra M.I.A., Jannah S.M., Chairani I. (2024). Therapeutic activities of honey in wound care: A narrative review. IOP Conf. Ser. Earth Environ. Sci..

[33] McLoone P., Oladejo T.O., Kassym L., McDougall G.J. (2024). Honey Phytochemicals: Bioactive Agents With Therapeutic Potential for Dermatological Disorders. Phytotherapy Res..

[34] Al-Kafaween M.A., Hilmi A.B.M., Al-Jamal H.A.N. (2022). Physicochemical and Therapeutic Properties of Malaysian Stingless Bee Kelulut honey in Comparison with Yemeni Sidr Honey. Anti Infect. Agents.

[35] Spoială A., Ilie C.-I., Ficai D., Ficai A., Andronescu E. (2023). Synergic Effect of Honey with Other Natural Agents in Developing Efficient Wound Dressings. Antioxidants.

[36] Sarheed O., Debe M.S. (2020). Honey Products and Their Potential in Wound Healing. Therapeutic Applications of Honey and Its Phytochemicals.

[37] Edros R.Z., Hamzah N.A., Shahlan A. (2019). Antibacterial Properties of Kelulut, Tualang and Acacia Honey Against Wound-Infecting Bacteria. Pertanika J. Trop. Agric. Sci..

[38] Azam N.S.M., Soh N.C., Rapi H.S., Ismail N., Jusoh A.Z., Haron M.N., Ali A.M., Maulidiani M., Ismail W.I.W. (2022). In vivo study of subacute oral toxicity of Kelulut honey. Int. Food Res. J..

[39] Tomić S.L., Vuković J.S., Radić M.M.B., Filipović V.V., Živanović D.P., Nikolić M.M., Nikodinovic-Runic J. (2023). Manuka honey/2-hydroxyethyl methacrylate/gelatin hybrid hydrogel scaffolds for potential tissue regeneration. Polymers.

[40] Lahooti B., Khorram M., Karimi G., Mohammadi A., Emami A. (2016). Modeling and optimization of antibacterial activity of the chitosan-based hydrogel films using central composite design. J. Biomed. Mater. Res. Part A.

[41] Masri S., Maarof M., Aziz I.A., Idrus R., Fauzi M.B. (2023). Performance of hybrid gelatin-PVA bioinks integrated with genipin through extrusion-based 3D bioprinting: An in vitro evaluation using human dermal fibroblasts. Int. J. Bioprinting.

[42] Salleh A., Mustafa N., Teow Y.H., Fatimah M.N., Khairudin F.A., Ahmad I., Fauzi M.B. (2022). Dual-Layered Approach of Ovine Collagen-Gelatin/Cellulose Hybrid Biomatrix Containing Graphene Oxide-Silver Nanoparticles for Cutaneous Wound Healing: Fabrication, Physicochemical, Cytotoxicity and Antibacterial Characterisation. Biomedicines.

[43] Wu P., Fisher A., Foo P., Queen D., Gaylor J. (1995). In vitro assessment of water vapour transmission of synthetic wound dressings. Biomaterials.

[44] Uzun E.T., Gucu I., Arslan T., Kalkan S.O. (2019). Retrofitting of Masonry Structures Considering the Architectural Perspective: A Case Study in Foca, Izmir. IOP Conf. Ser. Mater. Sci. Eng..

[45] Kent M. (2016). Oxford Dictionary of Sports Science and Medicine.

[46] Wijianto B., Pratiwi L., Hermawati E. (2025). Evaluation of Physicochemical and Antioxidant Properties of Kelulut (*Heterotrigona itama*) Honey and Forest Honey Authentic to West Borneo. Lett. Appl. NanoBioscience.

[47] Abdel-Azim A.G., Abdel-Azim S.G., Abdel-Azim G. (2019). Determining the contribution of osmotic stress to the antibacterial properties of honey. Journal of Emerging Investigators. J. Emerg. Investig..

[48] Yusli E.R., Bachtia B.M., Suni D.F., Sutjiat A.B., Mozef T. (2016). Effect of Rambutan-honey and its Flavonoid on TGF-β1 Induce Fibroplasia Oral Wound Healing. Res. J. Med. Plant.

[49] Osato M.S., Reddy S.G., Graham D.Y. (1999). Osmotic effect of honey on growth and viability of Helicobacter pylori. Dig. Dis. Sci..

[50] Datta S., Sarkar R., Vyas V., Bhutoria S., Barui A., Chowdhury A.R., Datta P. (2018). Alginate-honey bioinks with improved cell responses for applications as bioprinted tissue engineered constructs. J. Mater. Res..

[51] Mahmoudi C., Douma N.T., Mahmoudi H., Tincu C.E.I., Popa M., Hamcerencu M., Andrițoiu C.V. (2024). Developing and characterizing a biocompatible hydrogel obtained by cross-linking gelatin with oxidized sodium alginate for potential biomedical applications. Polymers.

[52] Weng T., Zhang W., Xia Y., Wu P., Yang M., Jin R., Xia S., Wang J., You C., Han C. (2021). 3D bioprinting for skin tissue engineering: Current status and perspectives. J. Tissue Eng..

[53] Arif M.M.A., Fauzi M.B., Nordin A., Hiraoka Y., Tabata Y., Yunus M.H.M. (2020). Fabrication of Bio-Based Gelatin Sponge for Potential Use as A Functional Acellular Skin Substitute. Polymers.

[54] Erdagi S.I., Ngwabebhoh F.A., Yildiz U. (2020). Genipin crosslinked gelatin-diosgenin-nanocellulose hydrogels for potential wound dressing and healing applications. Int. J. Biol. Macromol..

[55] Wei P., Chen W., Song Q., Wu Y., Xu Y. (2021). Superabsorbent hydrogels enhanced by quaternized tunicate cellulose nanocrystals with adjustable strength and swelling ratio. Cellulose.

[56] Agubata C.O., A Mbah M., A Akpa P., Ugwu G. (2021). Application of self-healing, swellable and biodegradable polymers for wound treatment. J. Wound Care.

[57] Yap S.K., Chin N.L., Yusof Y.A., Chong K.Y. (2019). Quality characteristics of dehydrated raw Kelulut honey. Int. J. Food Prop..

[58] Haron H., Talib R.A., Subramaniam P., Arifen Z.N.Z., Ibrahim M. (2022). A comparison of chemical compositions in Kelulut honey from different regions.. Malays. J. Anal. Sci..

[59] Mohd Kamal D.A., Ibrahim S.F., Kamal H., Kashim M.I.A.M., Mokhtar M.H. (2021). Physicochemical and Medicinal Properties of Tualang, Gelam and Kelulut honeys: A Comprehensive Review. Nutrients.

[60] Dhena R.B., Hafid A., Aini M., Ahmad B., Erizal E. (2020). Utilizing Alginate to Improve Elasticity and Moisture Balance of Polyvinyl Alcohol/Chitosan Hydrogel Wound Dressing. Mater. Sci. Forum.

[61] Yasin S.N.N., Said Z., Halib N., A Rahman Z., Mokhzani N.I. (2023). Polymer-Based Hydrogel Loaded with Honey in Drug Delivery System for Wound Healing Applications. Polymers.

[62] Lin N., Zuo B. (2021). Silk sericin/fibroin electrospinning dressings: A method for preparing a dressing material with high moisture vapor transmission rate. J. Biomater. Sci. Polym. Ed..

[63] Seow E.-K., Gan C.-Y., Tan T.-C., Lee L.K., Easa A.M. (2019). Influence of honey types and heating treatment on the rheological properties of glutinous rice flour gels. J. Food Sci. Technol..

[64] Liang Y., He J., Guo B. (2021). Functional hydrogels as wound dressing to enhance wound healing. ACS Nano.

[65] Sagan K.P., Andrysiak-Mamos E., Sagan L., Nowacki P., Małkowski B., Syrenicz A. (2020). Cushing’s Syndrome in a Patient with Rathke’s Cleft Cyst and ACTH Cell Hyperplasia Detected by 11C-Methionine PET Imaging—A Case Presentation. Front. Endocrinol..

[66] Pope E.A., Roberts M.W., Johnson E.L., Morris C.L. (2018). Infant Oral Mutilation. Case Rep. Dent..

[67] Mitchell K., Panicker S.S., Adler C.L., O’toole G.A., Hixon K.R. (2023). Antibacterial Efficacy of Manuka Honey-Doped Chitosan-Gelatin Cryogel and Hydrogel Scaffolds in Reducing Infection. Gels.

[68] Masutani E.M., Kinoshita C.K., Tanaka T.T., Ellison A.K.D., Yoza B.A. (2014). Increasing Thermal Stability of Gelatin by UV-Induced Cross-Linking with Glucose. Int. J. Biomater..

[69] Lai J.-Y., Ma D.H.-K., Lai M.-H., Li Y.-T., Chang R.-J., Chen L.-M. (2013). Characterization of Cross-Linked Porous Gelatin Carriers and Their Interaction with Corneal Endothelium: Biopolymer Concentration Effect. PLoS ONE.

[70] Annabi N., Nichol J.W., Zhong X., Ji C., Koshy S., Khademhosseini A., Dehghani F. (2010). Controlling the porosity and microarchitecture of hydrogels for tissue engineering. Tissue Eng. Part B Rev..

[71] Gadge A.S., Shirsat D.V., Soumia P.S., Pote C.L., Pushpalatha M., Pandit T.R., Dutta R., Kumar S., Ramesh S.V., Mahajan V. (2024). Physiochemical, biological, and therapeutic uses of stingless bee honey. Front. Sustain. Food Syst..

[72] Gomes A., Teixeira C., Ferraz R., Prudêncio C., Gomes P. (2017). Wound-healing peptides for treatment of chronic diabetic foot ulcers and other infected skin injuries. Molecules.

[73] Razif R., Fadilah N.I.M., Ahmad H., Hao D.L.Q., Maarof M., Fauzi M.B. (2025). Asiaticoside-Loaded multifunctional bioscaffolds for enhanced hyperglycemic wound healing. Biomedicines.

